# Are Nursing Home Preferred Networks Good for Patients' Outcomes? Evidence From the Veterans Health Administration

**DOI:** 10.1093/geroni/igab046.074

**Published:** 2021-12-17

**Authors:** Portia Cornell, Emily Corneau, Kate Magid, Patience Moyo, James Rudolph, Cari Levy, Vincent Mor

**Affiliations:** 1 Providence VA Medical Center, Providence, Rhode Island, United States; 2 Rocky Mountain VA Medical Center, Aurora, Colorado, United States; 3 Brown University School of Public Health, Providence, Rhode Island, United States; 4 Brown University, Providence, Rhode Island, United States

## Abstract

In the Veterans’ Administration (VA), medical centers contract with community nursing homes to provide care to Veterans. As a purchaser, the VA could pursue a strategy of selecting a high-quality network; alternatively, it could focus resources on oversight by its nursing-home coordinators. The question of whether narrow networks are good for Veterans’ outcomes, conditional on quality, therefore, needs empirical investigation. We examined the effect of network concentration on hospital admissions, conditional on Veterans’ clinical acuity. We operationalized network concentration as the number of Veterans already in residence at the time of admission, and controlled for publicly reported quality measure (star rating). We identified 93,805 VA-paid admissions to nursing homes between 2013 to 2016. To address selectin bias, we estimated effects using a distance- based instrumental variable (IV) for each measure, with the log of distance to the nearest nursing home with a specified number of Veterans at the facility in the previous month (1-4, 5-9, and 10-13, and 14+ Veterans). Going to a facility with 10-13 or 14+ Veterans had a higher hospitalization probability (6.2 and 3.3 percentage points higher, respectively), than going to a facility with 1-4 Veterans. If quality rating improves outcomes, then broader networks are beneficial if consumers (Veterans) choose based on quality, given a broader choice set. Conditional on quality, concentrated networks do not seem to lead to fewer hospital admissions. Our results suggest that the VA could do more in its oversight role to work with these nursing homes to decrease hospital admissions.

